# Frontiers in therapeutic development of allopregnanolone for Alzheimer’s disease and other neurological disorders

**DOI:** 10.3389/fncel.2014.00203

**Published:** 2014-07-30

**Authors:** Ronald W. Irwin, Christine M. Solinsky, Roberta Diaz Brinton

**Affiliations:** ^1^Department of Pharmacology and Pharmaceutical Sciences, Pharmaceutical Sciences Center, School of Pharmacy, University of Southern CaliforniaLos Angeles, CA, USA; ^2^Clinical and Experimental Therapeutics Program, School of Pharmacy, University of Southern CaliforniaLos Angeles, CA, USA; ^3^Department of Neurology, Keck School of Medicine, University of Southern CaliforniaLos Angeles, CA, USA

**Keywords:** allopregnanolone, Alzheimer’s disease, β-amyloid, neurogenesis, regeneration, cholesterol homeostasis, myelin, treatment regimen

## Abstract

Allopregnanolone (Allo), a neurosteroid, has emerged as a promising promoter of endogenous regeneration in brain. In a mouse model of Alzheimer’s disease, Allo induced neurogenesis, oligodendrogenesis, white matter generation and cholesterol homeostasis while simultaneously reducing β-amyloid and neuroinflammatory burden. Allo activates signaling pathways and gene expression required for regeneration of neural stem cells and their differentiation into neurons. In parallel, Allo activates systems to sustain cholesterol homeostasis and reduce β-amyloid generation. To advance Allo into studies for chronic human neurological conditions, we examined translational and clinical parameters: dose, regimen, route, formulation, outcome measures, and safety regulations. A treatment regimen of once per week at sub-sedative doses of Allo was optimal for regeneration and reduction in Alzheimer’s pathology. This regimen had a high safety profile following chronic exposure in aged normal and Alzheimer’s mice. Formulation of Allo for multiple routes of administration has been developed for both preclinical and clinical testing. Preclinical evidence for therapeutic efficacy of Allo spans multiple neurological diseases including Alzheimer’s, Parkinson’s, multiple sclerosis, Niemann-Pick, diabetic neuropathy, status epilepticus, and traumatic brain injury. To successfully translate Allo as a therapeutic for multiple neurological disorders, it will be necessary to tailor dose and regimen to the targeted therapeutic mechanisms and disease etiology. Treatment paradigms conducted in accelerated disease models in young animals have a low probability of successful translation to chronic diseases in adult and aged humans. Gender, genetic risks, stage and burden of disease are critical determinants of efficacy. This review focuses on recent advances in development of Allo for Alzheimer’s disease (AD) that have the potential to accelerate therapeutic translation for multiple unmet neurological needs.

## Introduction

Neurosteroids, including allopregnanolone (Allo), are a class of neural messengers that regulate multiple processes in brain from ion channel properties to systems regeneration. Therapeutically it is possible to develop neurosteroid analogs to selectively target one action or to use endogenous neurosteroid molecules as systems biology regulators. Each strategy has its strengths and weaknesses. Depending upon the targeted disease and mechanism therein, it is possible to selectively activate a subset of responses or the full complement of potential responses. Therapeutic application of neurosteroids to achieve efficacy in a disease state requires both the understanding of the disease and the systems pharmacology of the neurosteroid (Brinton, 2013).

This review, while largely focused on therapeutic development of Allo for Alzheimer’s disease (AD), raises issues that are applicable more broadly to other diseases for which Allo may have therapeutic benefit. Thus the review is structured around a framework of *considerations that matter*. To substantially advance translational research that is predictive of clinical outcome, we address multiple issues that will ultimately determine translational feasibility and success of Allo across multiple disease conditions. Lastly, most neurodegenerative diseases have no cure although in some instances palliative care extends life but not function. Therapeutics to prevent neurodegenerative disease in populations at risk, delay progression of disease in those affected and restore function in those with late stage disease remain elusive. It is our goal to enable others in the field to advance with greater speed and greater success by considering key aspects of translational research that really matter.

## Molecular pharmacology matters

Mechanisms of drug action should be optimally identified, confirmed and characterized in preclinical studies before progressing to clinical trials (Becker et al., 2014). Metabolites of progesterone with reduced A-ring steroid structures are potent endogenous agonist modulators of gamma-aminobutyric acid (GABA) type A receptors (GABA_A_Rs). The GABA_A_R is a ligand-gated ion channel and is primarily associated with inhibition of or fine-tuning of excitatory neurotransmission. The neurosteroid progesterone metabolites that modulate the GABA_A_R include 3α-hydroxy-5β-pregnan-20-one (pregnanolone); 3α-hydroxy-5β-pregnan-20-one (allopregnanolone; Allo; 3α,5β-tetrahydroprogesterone) and its 21-hydroxylated derivative: tetrahydro-DOC (THDOC) derived from A-ring reduction of deoxycorticosterone. Of these metabolites, Allo is amongst the most potent endogenous allosteric modulators of the GABA_A_R (Belelli and Lambert, 2005). Within the mammalian brain, Allo has been shown to modulate anxiety, depression, seizure activity, sedative-hypnotic activity, and the immune system. Adding to this list, we have shown that Allo promotes the neuroregenerative system and modifies the course of neurodegenerative disease.

Neurosteroid molecular structures and their structure-activity relationship with their cognate receptors have undergone eons of co-evolutionary selection. Steroid binding site-containing GABA_A_Rs could have evolved during early chordate evolution, possibly between the branch points of Cephalochordata (lancelets) and Agnatha (lampreys) (Paul and Purdy, 1992). Much earlier, phylogenetic studies conclude that ligand gated ion channels evolved from protoreceptors in unicellular organisms (Pierobon et al., 2004).

Ionotropic GABA_A_Rs primarily transport chloride and bicarbonate ions. The potencies and efficacies of neurosteroids including Allo depend on the subunit composition of GABA_A_Rs. GABAergic neurotransmission can be fine-tuned by allosteric modulation at GABA_A_R binding sites for barbiturates, benzodiazepines, anesthetic alcohols, and neurosteroids. At low concentrations, neurosteroids bind to GABA_A_R at distinct sites to act as positive or negative modulators of GABA_A_R function (Gee et al., 1987, 1988). Allo is a potent positive allosteric activator of GABA_A_R channels that at nanomolar concentrations enhances the apparent affinity of GABA for GABA_A_R and, at micromolar concentrations, can directly activate GABA_A_R chloride channels. Allo binds to two transmembrane sites of the heteropentameric GABA_A_R assembled from eight subunit families (Hosie et al., 2006). GABA_A_R binding sites have the general subunit stoichiometry 2α:2β:1γ. GABA_A_R channel complexes that occur at the synaptic cleft have a higher threshold for activation and display phasic conductance. In contrast to synaptic GABA_A_Rs, a subset of extrasynaptic GABA_A_Rs, contain the neurosteroid-sensitive δ subunit making them pharmacologically distinct and display a tonic conductance pattern (Meldrum and Rogawski, 2007). GABA_A_Rs have been identified by electron microscopy in adult hippocampal subgranular zone (SGZ) progenitor cells (Mayo et al., 2005). Surrounding local interneurons that project towards the neurogenic niche and release GABA to adult dentate granule cells are subjected to tonic GABAergic signaling via δ-subunit-containing GABA_A_Rs (Overstreet Wadiche et al., 2005). Functionally, GABA plays a key role in the generation of spontaneous network activity within immature dentate granule cells (Owens and Kriegstein, 2002; Sipila et al., 2004).

GABAergic signaling likewise controls proliferation of adult progenitor cells within the subventricular zone (SVZ) neurogenic niche (Liu et al., 2005). Progenitor cells in the SVZ co-express GABA_A_R β_2_, β_3_ receptor subunits, GAD65/67 and GFAPδ (Dieriks et al., 2013). The expression of GAD65/67 was detected at lower amounts in the SVZ than in the caudate nucleus, and co-labeling was observed with GABA_A_R β_2_, β_3_, and PCNA, suggesting that cells with these markers utilized GABA from early neurogenesis until maturity. GABA_A_R γ_2_ was the most abundant and highly localized to the SVZ. GABA_A_Rs are found throughout the SVZ on all major cell types, however GABA_A_R γ_2_ shows the highest specific expression in the SVZ (Dieriks et al., 2013).

Through a PKC-dependent signaling mechanism, the neurosteroid THDOC selectively potentiated phosphorylation and membrane insertion of the α_4_/δ subunit-containing extrasynaptic GABA_A_R subtypes mediate tonic conductance in the dentate gyrus (Abramian et al., 2014). This effect of THDOC was specific as it did not phosphorylate α_5_/δ or KCC2 (Abramian et al., 2014).

The mechanism of action for Allo activated cell cycle gene expression in neural stem cells is mediated by binding to GABA_A_R to elicit an efflux of chloride and a concomitant influx of calcium that contributes to the induction of cell signaling events which lead to gene transcription of mitotic genes and downregulation of anti-mitotic genes (Brinton, 2013). A rapid rise in intracellular calcium, and subsequent activation of the cell cycle, initiates neurogenesis (Wang et al., 2005; Wang and Brinton, 2008; Brinton, 2013). Upon exogenous administration, a threshold brain concentration of Allo in neural progenitor cells of the neurogenic niches, activates a signaling cascade to trigger cell proliferation (Wang et al., 2005; Wang and Brinton, 2008; Brinton, 2013). Allo in blood and brain is subsequently enzymatically cleared within a timeframe of minutes to hours (Zhu et al., 2001; Timby et al., 2006; Irwin and Brinton, 2014) sufficient to allow regenerative system required for Allo-induced neurogenesis to continue (Brinton, 2013).

Collectively these data provide a window into the richness of the GABA_A_R system and how Allo modulates the function of these receptors to affect both the excitability of the brain and its regenerative capacity. The unanticipated link of the GABA_A_R to the regeneration of neural stem/progenitor cells suggests the possibility that other unanticipated and exciting relationships have yet to be identified.

## Cholesterol and the steroidogenic system matter

The brain must synthesize its own supply of cholesterol from acetyl-CoA independent of peripherally circulating cholesterol. Because all steroids are generated from cholesterol, changes in cholesterol homeostasis will inevitably affect steroidogenesis.

Cholesterol must be delivered to and from cells by lipoproteins which include low-density lipoproteins (LDL) and high-density lipoproteins (HDL). The lipoprotein component ApoE, shuttles cholesterol between cells through the interstitial fluid and therefore regulates the distribution and redistribution of cholesterol to each cell type (Mahley, 1988). Cholesterol is transported within the cell via steroidogenic acute regulatory protein (StAR) to the mitochondrial membrane translocator protein (TSPO). TSPO is a mitochondrial rate-limiting control checkpoint regulating cholesterol uptake and thus the synthesis of neuroactive steroids (Rupprecht et al., 2010; Irwin and Brinton, 2014). TSPO forms a cholesterol transport pore in the mitochondrial inner membrane with other proteins that include the StAR, voltage-dependent anion channel protein (VDAC), and adenine nucleotide transporter protein (ANT).

The cholesterol transport pore transports cholesterol to the mitochondrial matrix to be converted into pregnenolone by the cytochrome P450 side-chain cleavage (CYP450scc) enzyme (Liu et al., 2006). Pregnenolone diffuses out of the mitochondrial compartment and is converted in the cytosol to progesterone by 3β-hydroxysteroid dehydrogenase (3β-HSD). Two enzymes, 5α-reductase (5α-R) type-I and 5α-hydroxysteroid dehydrogenase (3α-HSD) are required to synthesize Allo from progesterone (Mellon et al., 2001; Mellon, 2007).

Allo is a reduced metabolite of progesterone, synthesized in the gonads, adrenal cortex, and the central nervous system (Genazzani et al., 2000). In the central and peripheral nervous systems, Allo synthesis occurs primarily in glial cells—astrocytes, oligodendrocytes, and Schwann cells and in many neuronal cell types including neural progenitors (Melcangi et al., 1996; Griffin and Mellon, 2001; Mellon and Vaudry, 2001; Benarroch, 2007).

An expression pattern of progesterone converting enzymes is evident in both hippocampus and cortex. Endogenous Allo production is controlled by the rate-limiting reduction of progesterone to 5α-dihydroprogesterone (5α-DHP) by 5α-R. Progesterone is converted to Allo by the sequential action of 5α-R type-I, to 5α-DHP, which is then converted by 3α-HSD to form Allo. 5α-reductase and 3α-hydroxysteroid dehydrogenase are functionally expressed in pluripotent progenitors, neural progenitor cells, and subsets of hippocampal neurons that contain 5α-R and 3α-HSD (Melcangi et al., 1996). Subsequently, 3α-HSD catalyzes conversion of 5α-DHP into Allo. Amyloid-β-binding alcohol dehydrogenase (ABAD) is an enzyme that is associated with mitochondria and facilitates back conversion of Allo to 5α-DHP (Yang et al., 2005). Interestingly, anti-depressants, such as fluoxetine, were demonstrated to increase Allo production and although not directly correlated, increased neurogenesis (Malberg et al., 2000; Uzunova et al., 2004, 2006).

Increasing evidence indicates that altered cholesterol homeostasis is linked to neuropathologies including AD (Schumacher et al., 2004; Mellon et al., 2008; Brinton, 2013). In addition to the mechanism of action whereby Allo induces neurogenesis (Brinton, 2013), Allo regulates cholesterol homeostasis via mechanisms that increases liver-X-receptor (LXR) and pregnane-X-receptor (PXR; Chen et al., 2011). LXR is a nuclear hormone receptor abundant in the brain, primarily expressed in glial cells and acts as a molecular sensor of cholesterol levels and initiates cholesterol clearance (Whitney et al., 2002; Jakobsson et al., 2012). Loss of either LXRα or LXRβ subtype expression exacerbated AD-related pathology in APP/PS1 double transgenic mice (Zelcer et al., 2007). Loss of LXR has been shown to repress cortical neurogenesis particularly during late-embryonic stage development of layer II/III (Fan et al., 2008). LXR activation increases cholesterol efflux through increased ABCA1 and ApoE expression, and prevents overactivation of γ-secretase and overproduction of Aβ (Whitney et al., 2002; Shenoy et al., 2004; Jiang et al., 2008). LXR activation improved cognitive function in multiple mouse models of amyloidogenesis (Schultz et al., 2000; Whitney et al., 2002; Yang et al., 2006; Xiong et al., 2008; Donkin et al., 2010; Leduc et al., 2010a).

LXRs are recruited to ABCA1 gene promoter regions and ApoE expression to decrease Aβ plaque formation and increase Aβ clearance (Koldamova et al., 2010) through phagocytosis by microglia (Terwel et al., 2011). Further, LXRs are recruited to the ABCG1 promoter in a ligand-dependent manner to alter epigenetic histone methylation allowing for a relaxed chromatin structure accessible to further gene expression and cholesterol efflux (Jakobsson et al., 2012). LXRs reduce neuroinflammation by inhibition of inflammatory genes (Zelcer et al., 2007). Inflammatory cytokines reach high levels in AD and when suppressed by LXR activation, enhance the phagocytic activity of microglia and thus Aβ clearance. LXR ligands activate PXR (Riddell et al., 2007). In parallel with an Allo-induced increase in LXR expression in the pre-pathology condition, Allo also increased PXR expression in the pre-pathology 3xTgAD mouse brain (Chen et al., 2011). PXR activation, primarily in neurons, induces cytochrome P450 3A (CYP3A) enzymes including CYP3A4 and CYP3A13 and subsequent cholesterol hydroxylation and activation of organic anion transporters (OATs) for cholesterol extrusion (Sun et al., 2003). In addition to increased LXR and PXR expression, Allo treatment initiated in pre-Aβ pathology 3-month-old 3xTgAD mice treated once per week for 6 months increased expression of 3-hydroxy-3-methyl-glutaryl-CoA-reductase (HMG-CoA-R) (Chen et al., 2011). Although HMG-CoA-R is the rate-limiting enzyme in cholesterol synthesis, it is also required for production of oxysterols that activate LXR and PXR-mediated gene transcription of cholesterol- and lipid-transport proteins (Leduc et al., 2010b). These data predict that an Allo-induced increase in brain LXR and PXR leads to increased cholesterol efflux, thereby reducing gamma-secretase activation by cholesterol-laden lipid rafts. Allo-stimulated cholesterol efflux is a plausible mechanism for the observed reduction of 27 kD and 56 kD intraneuronal Aβ oligomers after 6 months of once per week treatment (Chen et al., 2011).

*In vivo*, brain cholesterol homeostasis and intraneuronal Aβ are tightly coupled with Allo efficacy (Chen et al., 2011). Deposition of Aβ in the extracellular compartment disconnected this coupled pathway and led to a loss of Allo efficacy in advanced stages of AD-like pathology in the 3xTgAD model. Allo significantly reduced Aβ generation in hippocampus, cortex, and amygdala, which was paralleled by decreased mitochondrial ABAD and reduced microglia activation assessed as reduced expression of Iba-1 (Chen et al., 2011). A reduction in ABAD expression lowers mitochondrial dysfunction and simultaneously decreases the amount of Allo that is enzymatically back-converted to its precursor steroid 5αDHP. Allo may stimulate oligodendrocyte progenitor cells in addition to neural progenitor cells and since Allo is a metabolite of progesterone, the observed increases in oligodendrogenesis with progesterone treatment could be due to Allo (Schumacher et al., 2012). The myelin marker CNPase, a myelination marker, was increased by once per week Allo, indicating myelinating capabilities in the 3xTgAD mouse model (Chen et al., 2011). In the pre-Aβ pathology cohort, 3-month-old 3xTgAD mice displayed increased expression of liver-X-receptor, pregnane-X-receptor, and 3-hydroxy-3-methyl-glutaryl-CoA-reductase (HMG-CoA-R), three key proteins that regulate cholesterol homeostasis (Chen et al., 2011). Collectively, Allo is a systems biology regulator that promotes the neuroregenerative system with a simultaneous reduction of AD pathology in the 3xTgAD male mouse model.

## Neurosteroid formulation matters

The physico-chemical properties of Allo create a challenge for aqueous formulation. Allo’s low molecular weight (318.49 g/mol) and low number of hydrogen bond donors (one) and acceptors (two) are advantageous brain-targeting properties. However, the logP-value for Allo, 5.042, poses a solubility challenge for aqueous formulation and thus hinders its use as an orally administered drug (Luchetti et al., 2010). Further challenges exist for enteral absorption of Allo after absorption. To avoid the issues with Allo absorption through the oral route, Allo formulations were developed for parenteral routes of administration.

**Figure 1:**
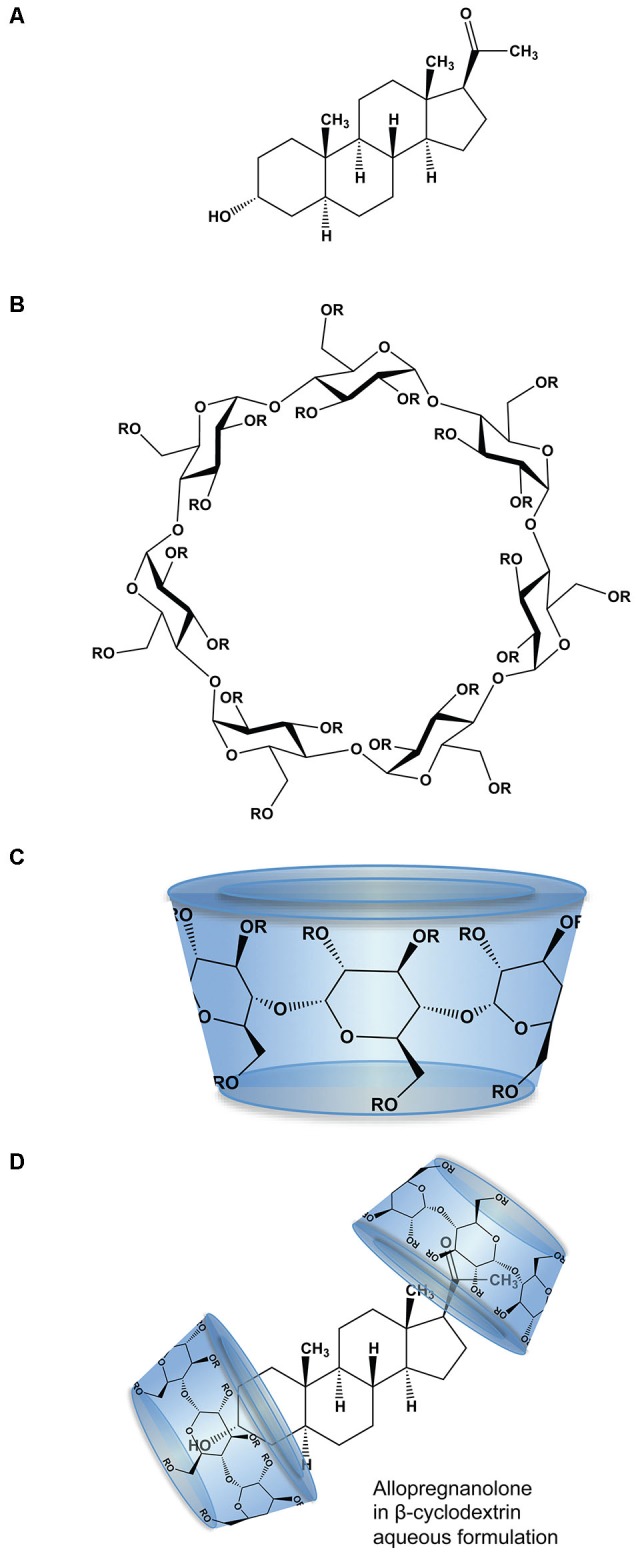
**Molecular structure and formulation of allopregnanolone**. **(A)** Chemical structure of allopregnanolone; molecular weight 318.49 g/mol. **(B)** Chemical structure of β-cyclodextrin derivatives, where R is hydrogen or 2-hydroxypropyl (CH_2_CHOHCH_3_) for HBCD or sulfobutylether ((CH_2_)_4_ SO_3_ Na) for SBECD. HBCD has an average molecular weight ~1460 g/mol, with 0.7–3.15 average degrees of substitution for hydrogen. SBECD molecular weight ~2163 g/mol with average degrees of substitution estimated between 6.3–6.6. **(C)** The β-cyclodextrins form three-dimensional cyclic oligosaccharide toroidal structures comprised of 1,4-linked glucopyranose molecules with a hydrophilic outer surface and a hydrophobic inner surface. **(D)** Allopregnanolone in β-cyclodextrin formulation to render allopregnanolone water soluble for rapid release *in vivo*. Two or more molecules of β-cyclodextrin, represented as toroid-shaped hydrophilic caps, form an inclusion complex with each relatively insoluble Allo molecule.

The physico-chemical properties of SBECD aid in solubilization of drug molecules with low aqueous solubility including Allo. SBECD is a chiral molecule composed of 7 α-D glucopyranose units with a molecular weight of approximately 2163 (molecular formula C_42_H_70–n_O_35_•(C_4_H_8_SO_3_Na)_n_•xH_2_O [*n* = ~6.6]; Figure 1). As does its parent molecule, 2-hydroxypropyl β-cyclodextrin (HBCD; HPBCD), sulfobutylether β-cyclodextrin (SBECD) has a primary face diameter of 7.8 Å (or 0.78 nanometers) and a secondary face diameter of 15.3 Å (or 1.53 nanometers). The SBECD chemical structure differs in the side chain hydroxyl groups of HBCD, replaced by sulfo-butyl-ethers thus improving its solubility properties. Multiple SBECD molecules surround each Allo molecule to enable aqueous solubility and enhance delivery properties (Figure 1).

The pharmacokinetics of SBECD revealed a low volume of distribution (Vd) corresponding to extracellular water and a short elimination half-life (t_1/2_) (Luke et al., 2010). Renal clearance of SBECD was at a rate corresponding to the glomerular filtration rate in all species investigated. A single IV dose of 600 mg/kg SBECD was administered to male mice. Clearance and Vd were 20.5 mL/min/kg and 0.98 L/kg, respectively; the t_1/2_ was 0.6 h (Luke et al., 2010). In the rabbit, clearance was 5.5 mL/min/kg, Vd was 0.24 L/kg, and t_1/2_ was 0.5 h. A single IV dose of 240 mg/kg SBECD in the dog resulted in a clearance of 4.7 mL/min/kg, Vd of 0.43 L/kg, and t_1/2_ of 1.1 h (Luke et al., 2010). No clinical evidence of toxicity was found in dogs at daily doses up to 1500 mg/kg (Luke et al., 2010). The no observed adverse event level (NOAEL) for IV SBECD, associated with vacuole uptake of renal proximal tubule epithelium, was set at 80 and 30 mg/kg in rats and dogs, following bolus injection. The NOAEL associated with foamy or lipid-laden macrophages in the lung was established at 160 mg/kg in rats and 200 mg/kg in dogs. SBECD is renally excreted intact and in all studies, no evidence of metabolism of SBECD exists (Luke et al., 2010).

SBECD is approved for use in marketed drug products including intravenous voriconazole, amiodarone, ziprasidone, aripiprazole, and maropitant (Luke et al., 2010). Human exposure data based on Pfizer’s regulatory submission were derived from four clinical studies where IV SBECD was administered (Luke et al., 2010). A total of 49 healthy male volunteers received IV infusions of SBECD alone. SBECD doses between 25 and 200 mg/kg/day were used to assess the safety and pharmacokinetics. In patients with renal problems, steady-state conditions indicated that even with daily hemodialysis, SBECD was effectively eliminated during 6 h of renal replacement therapy (Hafner et al., 2010). In older patients such as those in AD clinical trials, renal function is an important consideration when selecting a formulation and monitoring its safety and clearance. SBECD is relatively safe and with intermittent exposure is unlikely to accumulate based on human pharmacokinetic studies.

The therapeutic dose of Allo for humans is likely within the dose-range explored in previous clinical studies with IV Allo 0.05–0.9 mg/kg (Timby et al., 2006; van Broekhoven et al., 2007; Grant et al., 2008; Kask et al., 2008, 2009). With a 30% SBECD formulation of Allo for example, the amount of SBECD would be less than the NOAEL with species allometric scaling. Hypothetically, IV Allo at 0.9 mg/kg or 6.3 mg/70 kg human, in a 5 mg/ml soluble formulation of 30% SBECD, would amount to 378 mg of SBECD or 5.4 mg/kg. Citing the NOAEL of 160 mg/kg in rats (Luke et al., 2010), allometric scaling to humans would approximate to 27 mg/kg, whereas the dose in this hypothetical tolerable dose situation would be approximately 5.4 mg/kg SBECD to deliver 0.9 mg/kg Allo.

**Figure 2:**
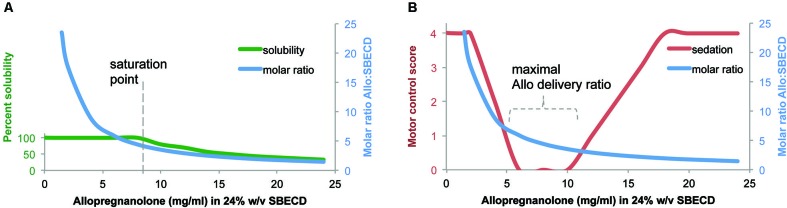
**Formulation of allopregnanolone matters**. **(A)** Percent solubility of allopregnanolone (Allo) in 24% w/v SBECD. Allo reached an optimal formulation of 6:1 at Allo 6 mg/ml in 24% SBECD. Solubility began to decline, observed by precipitation, when Allo concentration was between 8–10 mg/ml coinciding with a molar ratio of between 4–5 SBECD molecules per Allo molecule in water at room temperature without pH adjustment. **(B)** Motor control/sedation at 25 min after subcutaneous Allo 8 mg/kg to male rats measured by balance beam task on a five point scale where 4 = reaches platform; 3 = takes steps; 2 = all paws on top; 1 = clasp; 0 = fall off beam (Irwin et al., 2013). Molar ratio was plotted to illustrate relationship between Allo-induced loss of motor control and SBECD:Allo complexation ratio. Via subcutaneous injection, soluble formulations with SBECD:Allo between 7:1 and 3:1 maximally delivered Allo to systemic circulation resulting in rapid brain uptake observed by altered motor control. The cyclodextrin vehicle SBECD does not cross the blood brain barrier but facilitates Allo release to steroid carrier proteins in blood. The motor control/sedation test was used as a safety biomarker of maximally tolerable Allo target engagement in brain.

The complexation ratio of the combination of the excipient (SBECD) and the neurosteroid (Allo) has limitations based on solubility properties (Figure 1). The complexation ratio of Allo with cyclodextrins such as SBECD is a major determinant of release of Allo into the blood and brain (Figure 2). To develop formulations with clinical utility, we tested multiple Allo/SBECD complexation ratios on behavior in adult rats (Irwin et al., 2013). The optimal Allo:SBECD formulation (molar ratio of 5.89) was fully soluble and bioavailable as indicated by rapidly induced and prolonged sedation at the maximally tolerated dose for sedation 8 mg/kg subcutaneous in rats. When the ratio was increased (molar ratio of 23.56), the rate of Allo release into the brain was reduced as indicated by a lack of sedation (Figure 2B). Likewise, when the ratio was decreased (molar ratio of 1.47) relative to optimal, the rate of Allo release into the brain was also reduced (Figure 2B). The volume of soluble Allo administered in the saturated suspension formulation is limited when dosing 8 mg/kg to rats and results in mild sedative effects likely due to the relatively small fully soluble fraction giving the suspension formulation dual properties. Based on these data, it was postulated that a small but fully soluble fraction was rapidly delivered to the brain at a low dose followed by a slowly absorbed suspension fraction that does not possess the release rate required for sedation at the 8 mg/kg dose level (Irwin et al., 2013).

Efforts to synthesize water-soluble analogs of neurosteroids, including Allo and progesterone have been made with the goal to maintain structure-activity relationships (MacNevin et al., 2009). Excipients with prior regulatory approval are most desirable for formulations since they have undergone extensive toxicology testing and are more likely to gain regulatory acceptance when formulated with new active ingredients required to undergo their own extensive quality and safety assessments.

## Dose and route of administration matter

**Figure 3:**
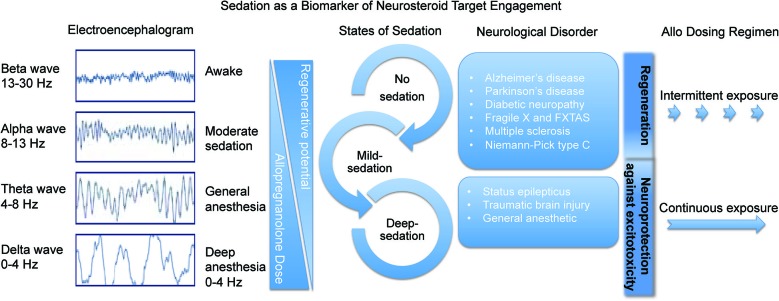
**Sedation as a biomarker of neurosteroid target engagement**. The sedative component of GABA_A_ receptor activation in the brain can be used a biomarker outcome of Allo delivery and tolerability. Through activation of the regenerative system in intermittent pulses, Allo is beneficial for many neurological disorders. In status epilepticus and traumatic brain injury it is important to deliver sedative doses of continuous Allo to protect the brain from further excitotoxicity.

For therapeutic use of Allo, it is imperative to determine the optimal dose, formulation, and dosing regimen (Brinton, 2013). The dose of Allo matters within the context of the targeted biological system. Safety and efficacy must be balanced to avoid unnecessary overexposure and remain consistent with a balanced neuroendocrine system (Figure 3). For most efficacy outcomes, Allo, like other neurosteroids, has an inverted U-shaped dose response profile—too high or too low of an Allo dose leads to suboptimal responses (Wang et al., 2005). Allo induces neurogenesis through potentiation of GABA_A_R chloride channels in neural progenitor cells in a dose-dependent manner: 10, 100, and 250 nM doses were efficacious whereas neurogenic efficacy diminished at higher doses (Wang et al., 2005; Wang and Brinton, 2008).

*In vivo*, we have shown that both dose and route matter to determine the therapeutic range and tolerability of Allo (Irwin et al., 2011; Irwin and Brinton, 2014). In our previous *in vivo* studies, subcutaneous Allo was administered in the range of 1–20 mg/kg to mice (Wang et al., 2010). The dose of Allo 10 mg/kg via subcutaneous route was selected for subsequent studies based on hippocampal BrdU incorporation as an indicator of neurogenesis (Wang et al., 2010). Subcutaneous Allo injections were also shown to be effective in a mouse model of Niemann-Pick Type C (NPC; Griffin et al., 2004) and in addition to increased myelin formation and decreased demyelination, the efficacy of Allo was partly due to a reduction of oxidative stress (Zampieri et al., 2009).

For human translation, subcutaneous or intramuscular injections are advantageous due to ease of administration, patient compliance, and tolerability. Allo pharmacokinetics and pharmacodynamics were determined by a combination of brain and blood levels associated with degree of sedation and amount of hippocampal neurogenesis (Irwin et al., 2013). Our recent studies found that sedation following subcutaneously administered Allo in the rat was more variable than the intramuscularly administered response (Irwin et al., 2013). Our analyses indicated that an intramuscular dose was approximately twice as potent at inducing sedation as subcutaneous delivered in a rapid release formulation (Irwin et al., 2013). The no-observable-adverse-effect or subsedative dose level was finely tuned by conducting a series of rat sedation studies within the dose range of our mouse efficacy studies. Subsedative doses were determined to optimally increase markers of neurogenesis including significant increases in BrdU labeled nuclei in the hippocampus (Irwin et al., 2013). Pharmacokinetic studies often begin with the intravenous route to provide maximal bioavailability and inform further studies by alternative routes. An intravenous Allo no-observable-adverse-effect dose of 0.5 mg/kg in mice or 0.2 mg/kg to rats is predicted by allometric species conversion calculations to be equivalent to a human intravenous dose of approximately 0.42 mg/kg or ~3 mg for a 70 kg adult. Allo has been tested in preclinical models via multiple routes of administration to establish safe dosage ranges for each route.

When selecting an Allo dose or reviewing the available literature (Irwin et al., 2011; Irwin and Brinton, 2014), it is important to compare and contrast the treatment methods and animal models within each study. Evaluation of therapeutic potential requires a deep understanding of the dose and route of administration that will influence pharmacokinetic and pharmacodynamic outcomes.

## Treatment regimen matters

### Alzheimer’s disease

Neurogenesis occurs over the course of hours in the case of transitioning through the cell cycle, to months in the case of full integration into hippocampal circuitry and networks (Brinton, 2013). Activating both regenerative and repair systems to maximize therapeutic benefit in the ageing or degenerating brain requires dual assessment of pathology and proliferation simultaneously. Further, multiple dosing regimens should be tested in order to conclude that regimen does matter and to determine an optimal therapeutic regimen. Our studies followed this plan to determine that Allo treatment regimen matters, testing administration of Allo every day, every other day, every week, and every month. Results of these studies indicate that a once per week treatment regimen with a single dose of Allo (10 mg/kg) was optimal to promote neurogenesis while also activating systems that reduced Alzheimer disease pathology (Chen et al., 2011).

We previously demonstrated that Allo induces neural stem cell cycle gene expression (Wang et al., 2005) and induces key regulators of cholesterol homeostasis (Chen et al., 2011) to provide mechanistic plausibility for its therapeutic efficacy to promote neurogenesis and cognitive function while reducing AD pathology following intermittent dosing. Allo promotes neurogenesis (Wang et al., 2005, 2010), recovery of learning and memory function (Wang et al., 2010; Singh et al., 2012), and reduction of AD pathology burden (Chen et al., 2011) in the 3xTgAD mouse model. We have demonstrated that regeneration is achieved with either once per month or once per week regimen of Allo (Chen et al., 2011). In the same mouse model, the reduction of AD pathology was achieved with once per week or every other day regimens (Chen et al., 2011; Irwin et al., 2011; Brinton, 2013). A combination of regeneration and reduction of pathology was achievable with an intermittent, once per week, Allo treatment regimen (Figure 3).

Intermittent Allo treatment regimens have shown benefit in a spectrum of preclinical neurodegenerative disease models including NPC (Griffin et al., 2004), diabetic neuropathy (Leonelli et al., 2007), peripheral nerve injury (Meyer et al., 2008), multiple sclerosis (Noorbakhsh et al., 2011), and Parkinson’s disease (Adeosun et al., 2012). Frequent or constant exposure to Allo is not better for regenerative processes although more often than once per week improved reduction of β-amyloid-burden disease-modifying effects (Chen et al., 2011). By contrast, a constant infusion treatment regimen over the course of months was anti-regenerative and resulted in adverse outcomes (Bengtsson et al., 2012, 2013; Irwin and Brinton, 2014). Unnecessary constant infusion of Allo is not biologically relevant and therefore should not be a therapeutic option for neurological disorders other than epileptic seizure and traumatic injury (Figure 3).

Allo concentrations in blood and brain are stress responsive and serve to restore normal GABAergic and hypothalamic-pituitary-adrenal (HPA) function following stress (Crowley and Girdler, 2014). An elevation of neurosteroids in response to stress is an adaptive anxiolytic response in acute stress situations. However, during chronic stress and depression, a condition collectively termed as allostatic load, system-wide decreases in brain and plasma neurosteroid concentrations occur and overall the response to acute stressors becomes dysfunctional (Genazzani et al., 1998; Bernardi et al., 2000; Dong et al., 2001; di Michele et al., 2003). Thus, a disruption in the biologic stress response system can exacerbate stress response disorders (Crowley and Girdler, 2014). Like the regenerative system, the stress response system requires a recovery period. Because of the temporal constraints of the regenerative system in brain, other regenerative factors are likely to have greater efficacy when administered with intermittent treatment regimens. In contrast, those emergency neurological conditions including traumatic brain injury and status epilepticus (Rogawski et al., 2013; Zolkowska et al., 2013) with intense seizure susceptibility and immediate risk of massive glutamate excitotoxicity and hypoxia require constant infusion regimens to dampen neuroexcitation to protect the central nervous system. We postulate that during the early stages of AD pathology development, administering pulsatile doses of Allo is therapeutically relevant to biological systems including the stress response system and the neuroregenerative system. Safe and tolerable Allo dose exposure serves as a neuroendocrine signal to initiate neuroregeneration.

Based on the therapeutic efficacy of Allo in a preclinical AD mouse model and in normal aged mice, we predict that Allo has potential therapeutic benefit in humans to delay progression in persons with familial early-onset AD and to prevent and delay disease in late onset AD. In these populations, Allo could be an effective therapy to promote the regenerative potential and myelination capacity of the brain to prevent or delay progression of mild cognitive impairment to clinically diagnosed AD. In summary, targeting a unique mechanism of action, Allo promotes the innate regenerative capability of the brain by increasing the number and survival of newly generated neurons. However, for therapeutic efficacy of Allo, it is imperative to determine the appropriate dosing regimen specific to each indication.

## Safety and tolerability matter

Earlier we reviewed the existing preclinical and clinical safety data in support of Allo therapeutic development (Irwin et al., 2011; Irwin and Brinton, 2014). Allo is a blood brain barrier penetrant molecule with previous safety data in humans (Timby et al., 2006; van Broekhoven et al., 2007; Grant et al., 2008; Kask et al., 2008, 2009). A cumulative dose of 0.9 mg/kg or approximately 6 mg for a 70 kg human, was administered to 9 men and 9 women with mean ages of 24.6 and 21.8 years respectively. Allo was generally well-tolerated with peak blood levels of 100 nM for women and 150 nM for men (van Broekhoven et al., 2007). Self-reported sedation and drowsiness were followed by and recovery followed the metabolic half-life of circulating Allo which was eliminated within hours (van Broekhoven et al., 2007). Physiological exposure to Allo is highest in the third trimester of pregnancy when levels reach 50 ng/ml or 157 nM (Luisi et al., 2000).

In human Phase 1 studies for AD, fully bioavailable intravenous injection of Allo in a dose escalation design will reach a limit with mild sedation to establish the maximally tolerated dose following intravenous administration. Currently, the upper physiological Allo blood concentration during the third trimester of pregnancy sets the safe exposure boundary until chronic toxicology studies conducted under Good Laboratory Practice (GLP) standards in rodent and non-rodent species are completed. Regulatory agencies require extensive and pivotal toxicology studies prior to large clinical trials and Allo drug product approval. These studies are necessary to demonstrate the safety within a specified dosage range and duration of exposure. GLP is a standardized quality system of management controls to ensure that in non-clinical laboratory studies the integrity of drug products such as Allo are planned, performed, monitored, recorded, reported and archived in a uniform, consistent, reliable, reproducible manner. GLP studies include extensive non-clinical safety tests that include specification of physicochemical properties generated under Good Manufacturing Practices (cGMP) and acute dose-range finding studies to chronic toxicity tests in rodents and non-rodent species to meet or exceed the duration of the clinical trial (Steinmetz and Spack, 2009).

In the US, all preclinical safety studies that contain investigational active pharmaceutical ingredients should seek regulatory guidance and oversight by the FDA. Regulatory agencies of many countries follow the International Conference on Harmonization guidelines. Pre-meeting with the regulatory agency will greatly improve important communication and a focused track to develop the Investigational New Drug (IND) application. IND approval in the US, or its equivalent application review process in other countries, must be obtained prior to initiation of standard clinical trials to establish safety and tolerability (Phase I) and efficacy (Phase II). Application for compassionate use IND requirements may be abbreviated for certain life threatening disease conditions outside of clinical trials that require emergency care decisions for expanded use discussed between physicians and regulatory agencies.

Safety reporting rules and timelines must also be maintained and monitored throughout the trial. In cases where existing preclinical toxicology and human exposure data are available, or highest physiological exposure has been studied, as is the case with Allo (Luisi et al., 2000), regulatory agencies may consider the active pharmaceutical ingredient to be sufficiently safe at a specified dosage range to move forward with safety and efficacy studies in humans. The entire process of drug development including time investment and funding must be kept in mind when planning viable therapeutic strategies for Allo. Along the Allo drug development timeline, leverage points are gained such as completion of chronic toxicology studies and should be shared when permissible to accelerate expanded access for neurological diseases similar dose, route, and treatment regimens overlap.

Toxicology studies define the safe exposure limits in animals and the outcomes are then extrapolated to predict maximally safe and tolerable exposure limits for initial human trials. Disease-modifying claims are stringently reviewed and may require additional safety studies beyond the standard toxicology battery. For example, recent AD beta-amyloid modifying therapies have shown in clinical studies to increase the risk for vascular edema, detected by magnetic resonance imaging (Salloway et al., 2009; Sperling et al., 2011b, 2012). Increased risk for microbleeds prompted regulatory agencies to require new safety measures for candidate AD drugs in pre-clinical stages. Drug candidates that claim to modify amyloidogenic mechanisms may require additional preclinical assessment of the associated risk for cerebral microhemorrhages in an appropriate animal model. Currently several AD mouse models are considered appropriate however these studies are costly in terms of time and money. Microhemorrhage risk assessment studies require cohorts of ~2 year old transgenic AD mice (Pfeifer et al., 2002; Racke et al., 2005; Demattos et al., 2012).

At the stage of dose range finding in rodents and non-rodents, tolerability predictions can be made ranging from the no-observed-adverse-effect level up to the maximally tolerated dose. For neurological disorders, a well-known biomarker of Allo target engagement and tolerability is sedation (Damianisch et al., 2001). Inhibition of the tuberomammillary nucleus has a key role in the sedative or sleep-inducing response to anesthetics that act on the GABA_A_R (Nelson et al., 2002). The sedation response to Allo can be objectively measured by detection of saccadic eye movement or brain activity via electroencephalogram (Figure 3; van Broekhoven et al., 2007) as was done with benzodiazepine drug development (Van Steveninck et al., 1993). Sedation level due to neurosteroids can also be subjectively determined by clinical observation and by visual analog scales (van Broekhoven et al., 2007). Typically, a combination of objective and subjective measures of sedation are used to assess tolerability. Allo induces dose-related sleep changes including a reduced sleep onset latency and increased pre-rapid-eye-movement sleep (Lancel, 1999). Allo has a safer tolerance profile than most GABA_A_R agonists including benzodiazepine hypnotics (Damianisch et al., 2001).

A neural network comprised of at least three discrete brain regions promotes sedation non-rapid eye movement sleep. GABAergic neurons of which Allo targets are in the ventrolateral preoptic nucleus are under tonic inhibition from noradrenergic neurons of the locus coeruleus. Inhibition of locus coeruleus neurons results in activation of the ventrolateral preoptic nucleus to induce sedation. GABAergic ventrolateral preoptic nucleus neurons of which Allo acts, innervate the ipsilateral tuberomammillary nucleus, a posterior hypothalamic cell group important in promoting arousal. The tuberomammillary nucleus, located on the ventrolateral edge of the posterior hypothalamus, contains neurons that co-express histamine and the inhibitory neurotransmitter GABA, and which project to the cerebral cortex, thalamus, and basal forebrain (Haas and Panula, 2003). These arousal-promoting, histaminergic tuberomammillary nucleus neurons are wake-active and are inhibited by the release of GABA and galanin by ventrolateral preoptic nucleus neurons. In short, the activated ventrolateral preoptic nucleus releases GABA to the GABA_A_R-containing sites of the tuberomammillary nucleus thus inhibiting release of arousal-promoting histamine into the cortex and forebrain to induce sedation. Through a wake-sleep neuronal network, at suprathreshold doses Allo allosterically potentiates GABA’s apparent affinity for the GABA_A_R to increase the chloride current of histaminergic neurons of the tuberomammillary nucleus causes sedation. The sedative response is not the primary regenerative target and sedation is less tolerable for treatment of chronic disease. A subsedative dose of Allo that retains activity at Allo-sensitive neural progenitor cells is optimal for regenerative responses and therapeutic development (Figure 3).

In a human clinical trial, a sedation-inducing dose of Allo briefly impaired episodic memory 10 min after the end of Allo intravenous infusion when peak blood levels were highest (Kask et al., 2008). Not surprisingly, a GABA_A_R allosteric agonist induced temporary memory impairment in a way similar to benzodiazepines. Chronic treatment paradigms that mimic stress conditions have also been shown to inhibit memory (Turkmen et al., 2006). Allo administered twice daily at high doses to male rats for several consecutive days decreased performance on the Morris water maze, escape latency, path length and thigmotaxis (Turkmen et al., 2006).

Collectively, the data indicate that the sedative properties of Allo are dose dependent and duration of exposure dependent. Allo administered by multiple routes of administration exhibits an acceptable margin of safety.

## Regenerative potential of the human brain matters

Most studies in AD mouse models, have reported decreased neurogenesis primarily in the hippocampal SGZ of the dentate gyrus and the SVZ lateral ventricles with association with cortical regions including the rostral migratory stream (Lazarov and Marr, 2010). There is strong evidence in AD animal models including the 3xTgAD mouse that demonstrate reduced neurogenesis with degree of AD pathology (Wang et al., 2007, 2010; Rodriguez et al., 2008, 2009; Chen et al., 2011; Singh et al., 2012). Unexpectedly, in post-mortem brain sections from AD victims, doublecortin, a microtubule-associated protein expressed by neuronal precursors and immature neurons, was increased relative to control brain sections (Jin et al., 2004). Neural progenitors have been isolated *in vitro* from post-mortem 11-week post-natal and adult human brain demonstrating that neural progenitors are present throughout life (Palmer et al., 2001). Further studies are required to determine whether the doublecortin immunostaining findings were reproducible with other antibodies or preferably with BrdU or ^14^C labels. It has been demonstrated that in humans without known neurological disorders, hippocampal neurogenesis occurs throughout adulthood and tapers modestly with advanced age (Spalding et al., 2013).

Humans exhibit substantial hippocampal neurogenesis within the SGZ (Spalding et al., 2013) and striatal neurogenesis associated with the SVZ lateral ventricles (Ernst et al., 2014). Humans, rather uniquely for mammals, do not demonstrate olfactory bulb neurogenesis, as the neuroblasts originating in the neurogenic niche of the lateral ventricle wall do not migrate to the olfactory bulb. Carbon-dated DNA within adult hippocampal neurons revealed that a substantial fraction of neurons were born during adulthood (Spalding et al., 2013; Ernst et al., 2014). Each year approximately 1.75% of the neurons turned over within the self-renewing fraction with only a modest decline during aging. A best-fit scenario model predicted that approximately 35% of the hippocampal cells were cycling corresponding to slightly less than the proportion that constitute the entire dentate gyrus region. From these studies it was estimated that the hippocampal dentate gyrus of human brain produces around 700 new neurons per day. Enough neurons could be replaced in the hippocampus to theoretically regenerate the entire hippocampal neurogenic region over the lifespan suggesting the importance of neurogenesis. Compared to rodents, humans may rely on neurogenesis even more during the aging process (Spalding et al., 2013). In healthy aging, the decline of hippocampal neurogenesis is less than the rate of decline when adjusted for lifespan and compared with rodent models.

Following results demonstrating hippocampal neurogenesis throughout the lifespan, Frisen and colleagues utilized the same ^14^C dating method to reveal abundant neuroblasts in the human striatum adjacent to the lateral ventricle wall and revealed a constant turnover/generation of striatal interneurons with and annual turnover rate of 2.7% within the renewing fraction (Ernst et al., 2014). This recent finding highlighted a major difference between human neurogenic niches vs. animal models. Rodents for example have well developed rostral migratory streams that send newborn SVZ neurons towards the olfactory bulb. Human brains were found to shunt the equivalent neurogenic niche cells to the striatum. In the brains of Huntington’s disease patients, a disease related to the degeneration of striatal neurons (Zuccato et al., 2010; Walker et al., 2011), the patient cohort in the advanced state of the disease lacked these post-natally generated neurons (Ernst et al., 2014).

Once new neurons develop, the cells require approximately 2 months to mature both morphologically and physiologically, suggesting to us that an intermittent treatment regimen (Figure 3) would be required to stimulate regeneration in this neurological disorder. The timing of therapeutic intervention must accommodate the development period from which newborn cells acquire GABAergic and glutamatergic inputs and receptors. During this development period the young neurons are highly excitable with increased synaptic dynamics compared to mature neurons (Gage and Temple, 2013). This time course of regeneration, migration, differentiation, creates a temporal map that is coincident with Allo-induced proliferation, increased learning capacity and increased memory function (Brinton, 2013).

## Gender matters

Neurological diseases that have varying prevalence, progression and severity between men and women include AD, Parkinson’s disease, attention deficit/hyperactivity disorders, and schizophrenia (Gillies and Mcarthur, 2010). Sex differences have been noted in human safety studies of Allo, where the same intravenous dose in men and women resulted in maximum blood levels that were higher in men 150 nmol/L vs. 100 nmol/L in women, although volume of distribution, elimination half-life, and the area under the curve (AUC) adjusted for body weight did not differ (van Broekhoven et al., 2007).

**Figure 4:**
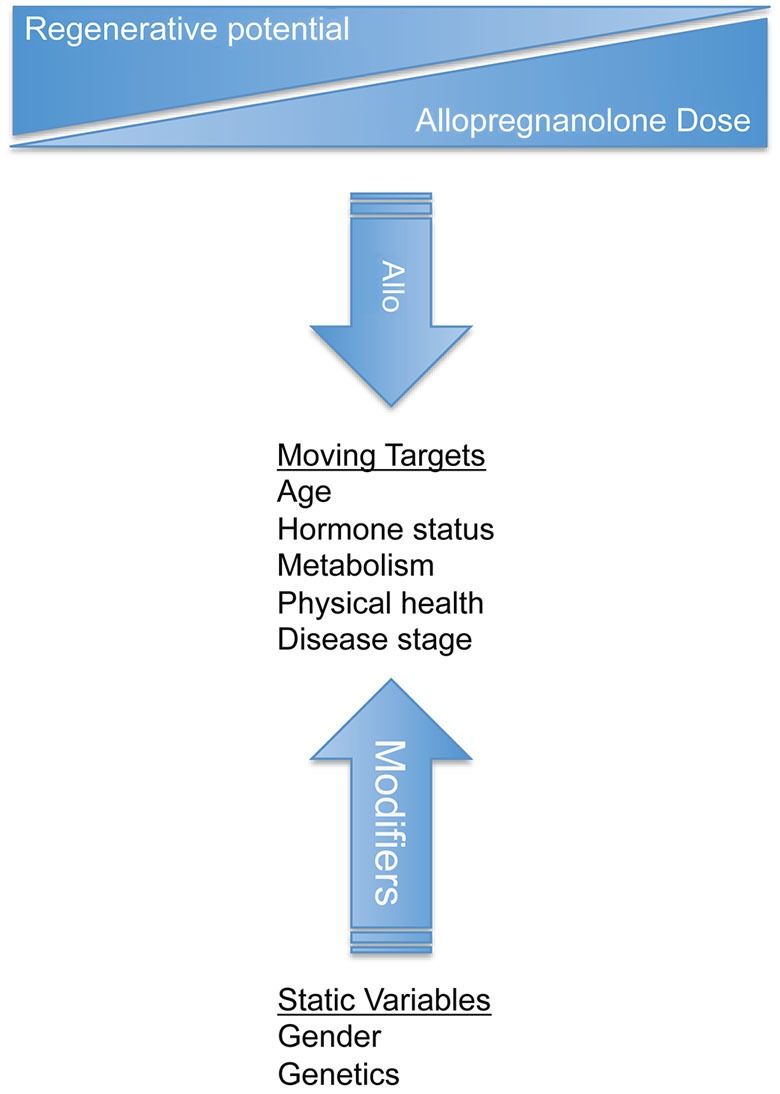
**Design of clinical trials for allopregnanolone should consider both static variables and moving targets to treat neurological disorders.** Gender and genetics are static variables that influence disease and response to treatment. Static variables impact the moving targets such as age, hormone status, metabolism, health, and disease progression. Successful clinical therapeutic strategies should be tailored for each disease state and should be monitored as the disease and treatment progresses. Dynamic biological processes require an understanding of disease system pathogenesis to select and modify the appropriate treatment regimen.

Clinical differences between females and males may result from differences in brain morphology, neurochemistry, and functional outcomes. It is hypothesized that there is a critical hormone treatment window in the post-menopausal brain. The importance of regimen is again demonstrated when it was shown that continuous vs. cyclic progesterone administration resulted in disparate gene expression profiles in the brain (Zhao et al., 2012). Changes in neural gene expression profiles after alternative progesterone therapies highlights the importance of mimicking physiological profiles of neurosteroid exposure in order to maintain and improve neurological health and function (Zhao et al., 2012). Changes in pathophysiology due to gender may be important in evaluating both efficacy and side-effect profiles during therapeutic development (Figure 4). Early in 2014, a drug response gender difference prompted the FDA to issue a safety announcement and reduce by half the zolpidem (Ambien) dosage for women compared to men.^1^ As a result, zolpidem is now the only prescription drug with a different dose for men and women. Zolpidem, like Allo, is a GABA_A_ agonist with sedative hypnotic actions in the wake-sleep neurocircuitry. Women metabolize and eliminate zolpidem slower than men, making them more susceptible to next morning impairment side effects.

Preclinically, a difference between sexes in Allo response was observed in our studies whereby male rats administered bolus injections of Allo had increased sensitivity to Allo’s sedative effects, with greater time and levels of sedation compared to age-matched female rats (Irwin et al., 2013). Allo may exert its effects on males and females differently, i.e., other than through binding postsynaptic GABA_A_Rs. The female brain may have a greater sensitivity to Allo’s potentiation of GABA neurotransmission. In a global ischemia mouse model, females were neuroprotected at a dose of Allo four times lower than the required dose to protect males (Kelley et al., 2011). These data are surprising in light of evidence indicating that male brains have a greater number of GABA_A_R binding sites relative to female brains (Juptner and Hiemke, 1990).

Cyclical changes of steroids during the estrous cycle can lead to changes in GABA_A_Rs and during late diestrus new receptors have α_4_, β_1_ and δ GABA_A_ receptor subunit expression (Lovick, 2008). As β1 subunit containing receptors are extrasynaptic and carry tonic currents, upregulation should lead to a decrease in GABAergic inhibition (Lovick, 2008). Studies in male rats have shown that alterations in Allo plasma levels can, within minutes, modulate the amount of GABAergic inhibition in the periaqueductal gray matter. Function changes resulting from altered intrinsic excitability levels may affect therapeutic responsiveness (Lovick, 2008).

Overall, there is a recognized need for a better understanding of sex differences related to neurotherapeutic response and the clinical relevance of such differences to inform and improve drug development.

## Disease target and therapeutic goals matter

### Alzheimer’s disease

#### Disease target

Regenerative system of the brain, cognition neural circuitry, and etiology of pathology.

#### Therapeutic response to Allo

Increased hippocampal neurogenesis, increase neural progenitor cell survival, and reverse learning and memory deficits, decreased microglial activation, and decreased amyloid-beta pathology (Brinton, 1994, 2013; Wang et al., 2010; Irwin et al., 2011; Singh et al., 2012; Lo et al., 2014).

#### Targeted mechanism of action

Potentiation of GABA at GABA_A_R to depolarize neural stem cells; regulation of LXR and PXR in cholesterol homeostasis.

#### Dose and treatment regimen

Intermittent exposure in mice: 10 mg/kg once weekly for 6 months (Chen et al., 2011); 1 mg/kg SC single dose, 10 mg/kg SC single dose, 20 mg/kg single dose (Wang et al., 2010); 10 mg/kg SC 3 times per week, for 3 months (Chen et al., 2011); 10 mg/kg once per month, single dose (Singh et al., 2012).

Allo and other trophic factors are decreased in blood and brain of AD patients compared to age-matched controls (Weill-Engerer et al., 2002; Marx et al., 2006; Naylor et al., 2010). Early AD is characterized by loss of episodic and semantic memory (Perry et al., 2000) and signifies hippocampal dysfunction. Diagnostic imaging studies using volumetric MRI revealed a decreased hippocampal volume due to neurodegeneration of gray matter in people diagnosed with amnestic mild cognitive impairment that will progress to AD (Whitwell et al., 2007). In AD, restoration of the dysfunctional neurogenic niche with intermittent pulses of Allo may regain neurological order while avoiding overstimulation.

We previously demonstrated a correlation between Allo-induced neural progenitor cell survival and improved memory function in the triple transgenic mouse model of AD (Brinton, 1994, 2013; Wang et al., 2010; Irwin et al., 2011; Singh et al., 2012). Consistent with the human AD brain neurosteroid profile (Weill-Engerer et al., 2002; Marx et al., 2006; Naylor et al., 2010), basal concentration of Allo in blood plasma of wild-type mice of was significantly lower than in cortex indicating higher brain accumulation of Allo. Higher level of brain Allo could be attributed to locally synthesized Allo in specific brain regions required for synaptic function. We also found that 3xTgAD mice had lower basal levels of Allo in the cerebral cortex (3xTgAD, 6.49 ± 2.02 ng/g vs. nonTg, 10.36 ± 1.43 ng/g), suggesting that there was either impairment of upstream Allo enzymatic production or accelerated Allo metabolism in 3xTgAD mice brain (Wang et al., 2010).

Within the SGZ and SVZ in male and female 3xTgAD mice a decline in neurogenesis is correlated with age-related AD-like pathology progression (Brinton and Wang, 2006; Rodriguez et al., 2008, 2009; Wang et al., 2010). Our studies have demonstrated that Allo promoted neurogenesis in the hippocampal SGZ to reverse learning and memory deficits (Wang et al., 2010). A study conducted in 3xTgAD mice subjected to an associative learning and memory task were analyzed and found increased neural progenitor cell survival of an intermittent treatment regimen with subcutaneous Allo 10 mg/kg, 3 weeks after a single Allo treatment and post-behavioral analyses. After 3 weeks, Allo-treatment vs. vehicle control demonstrated that surviving BrdU-labeled cells were located deep within the granular cell layer, consistent with the migration pattern of newly formed cells from the SGZ to the granule cell layer (Wang et al., 2010). In an *in vivo* dose-response study, 10mg/kg SC Allo exerted the greatest neurogenic efficacy and was the dose chosen for chronic preclinical efficacy assessment (Wang et al., 2010).

To further assess the preclinical efficacy of Allo for AD, our group has conducted studies of long-term intermittent exposure to Allo. These studies with Allo were also initiated with 3-months of age mice, prior to overt intraneuronal Aβ. In addition to neurogenic efficacy, these long-term studies were allowed us to determine the disease modifying effects afforded by the therapeutic regimen. Our group tested three treatment regimens—once per month, once per week, and every other day (Chen et al., 2011). Overall, we found that the optimal treatment paradigm with subcutaneous Allo, administered once-per-week for 6-months was maximally efficacious for both neurogenic and anti-Aβ endpoints (Importance of regimen discussed in greater detail above). Additionally, these studies demonstrated that together with the dosing frequency, the magnitude of pathology at the start of treatment intervention is critical to the window of therapeutic opportunity for Allo. Administration of Allo prior to and during the early stages of AD pathology significantly increased the regenerative response in brain while additionally reducing burden of pathology in an AD mouse model (Chen et al., 2011). In contrast, Allo treatment initiated at the point of Aβ plaque generation was not efficacious indicating that Allo targets regenerative and pathology reducing mechanisms present during the early to mid stages of the disease (Chen et al., 2011). After intraneuronal Aβ is extracellularly distributed, Allo’s efficacy becomes markedly diminished. Based on a collective body of preclinical data evaluating dose and route (Irwin et al., 2011; Brinton, 2013; Irwin and Brinton, 2014), we have selected the once per week regimen to move forward to FDA-compliant chronic toxicology studies and early phase clinical trials.

### Parkinson’s disease

#### Disease target

Regenerate dopaminergic system of the brain and etiology of pathology.

#### Therapeutic response to Allo

Neurogenesis in the substantia nigra, functional improvement in motor control (Adeosun et al., 2012), modulates dopamine release (Rouge-Pont et al., 2002).

#### Dose and treatment regimen

Intermittent exposure: 10 mg/kg once a week for 2 weeks in mice (Adeosun et al., 2012).

Clinical studies investigating the levels of endogenous Allo in PD patients demonstrated that in males, the levels of endogenous Allo were decreased both peripherally and in the CNS (di Michele et al., 2003, 2013). While Allo concentrations in specific human brain regions affected by PD has not been studied, in healthy women, the highest Allo levels were observed in the substantia nigra (SN) and basal hypothalamus (Bixo et al., 1997). The therapeutic goals for exogenous Allo are the modulation of dopamine release, restoration of neuroprotection, modulation of basal GABAergic tone, and neuroregeneration (Luchetti et al., 2010, 2011; Adeosun et al., 2012; di Michele et al., 2013). As in AD, the dose and therapeutic regimen must be carefully considered, as low doses Allo increases dopamine release, but at high doses decreases release (Rouge-Pont et al., 2002). In preclinical *in vivo* studies, once weekly Allo administration has demonstrated functional improvement (Adeosun et al., 2012).

### Multiple sclerosis

#### Disease target

White matter regeneration in the peripheral and central nervous systems, reduce inflammation, etiological mechanism of disease, and prevention of relapse.

#### Therapeutic response to Allo

Oligogenesis (Garay et al., 2012; Schumacher et al., 2014), neurogenesis (Ghoumari et al., 2003; Gago et al., 2004), cytoprotective, promyelination (Melcangi et al., 1999; Noorbakhsh et al., 2011), anti-inflammatory (Noorbakhsh et al., 2011), reduced disease severity (Noorbakhsh et al., 2011), innate immune function modulator (Noorbakhsh et al., 2014).

#### Dose and treatment regimen

Continuous exposure: daily IP 10 mg/kg for 28 days in mice (Melcangi et al., 1999). Intermittent paradigm: 1 mg SC every 4 days for 1 month in rats (Noorbakhsh et al., 2011).

The peripheral nervous system synthesizes steroid hormones, including progesterone, and possesses the enzymes required to convert these molecules to neuroactive metabolites, including Allo (Melcangi et al., 1999). In cases where the endogenous levels are insufficient, treatment with exogenous Allo could produce therapeutic benefit (Melcangi et al., 1999). Analyses of endogenous Allo showed that in MS patients, the Allo concentration in white matter was significantly decreased, demonstrating the potential for Allo as a potential therapeutic for this as yet unmet, clinical need (Noorbakhsh et al., 2011). A number of preclinical *in vivo* and *in vitro* studies have demonstrated the potential for Allo as a MS therapeutic through its anti-inflammatory effects, neurogenesis, and induction of myelin production (Melcangi et al., 1999; Ghoumari et al., 2003; Gago et al., 2004; Noorbakhsh et al., 2011). Common themes, seen in Allo studies for the disease states presented above, also apply to its utilization for MS. Efficacy is limited in late-stage disease, indicating that the system must retain neurogenic potential for therapeutic effect (Melcangi et al., 1999). Allo dosage and treatment regimen have been shown to be critical factors in study design. Positive effects of treatment were limited in *in vitro* studies when length of treatment was less than 24 h (Melcangi et al., 1999). As in AD, more Allo is not beneficial, and *in vitro* dose-response curve studies have demonstrated that the positive proliferative effects of Allo peak at 10 nM doses, and decrease with increasing doses (Gago et al., 2004).

### Niemann-pick type C

#### Disease target

Cholesterol trafficking and clearance system of the brain and etiology of disease.

#### Therapeutic response to Allo

Increased survival, decreased rate of motor control decline, increased neuronal survival, decreased cholesterol accumulation (Griffin et al., 2004), and reduced oxidative stress (Zampieri et al., 2009).

#### Dose and treatment regimen

Single dose exposure: 25 mg/kg SC, single injection in mice (Griffin et al., 2004). Continuous exposure: 0.5–2 mg in drinking water (ascending dose; lifetime treatment); 250 mg/90 days SC implant (lifetime treatment) in mice (Griffin et al., 2004).

NPC is a fatal, neurodegenerative, lysosomal storage disorder that affects cholesterol metabolism due to autosomal recessive mutations in the NPC1 and LPC2 loci. Allo has been studied for its therapeutic effect on NPC, which is characterized by defective trafficking of intracellular cholesterol and lysosomal accumulation of unesterified cholesterol gangliosides and other lipids leading to neurological deterioration and degenerating motor and cognitive function. Characterization of the NPC mouse model suggested dysfunctional steroidogenesis from cholesterol, giving credence to the indication for Allo (Griffin et al., 2004). *In vivo* studies have demonstrated that Allo, administered using continuous exposure paradigms results in improved outcomes, including survival and a decreased rate of decline of locomotion and motor coordination (Griffin et al., 2004). In addition to these functional improvements, histological examination demonstrated increased cerebellar neuron survival and decreased accumulation of cortical gangliosides (Griffin et al., 2004). Interestingly, in this disease state, continuous Allo administration via treated water resulted in superior survival outcomes vs. a single Allo SC dose. As has been shown in other disease states treated with Allo, timing of the single-dose injection is vital. Outcomes are best when Allo is administered early postnatally as efficacy diminishes and is quickly lost with advancing disease state (Griffin et al., 2004). *In vitro* studies have demonstrated a significant antioxidant function of Allo, but this remains to be translated to *in vivo* preclinical models (Zampieri et al., 2009).

### FXTAS and Fragile X syndrome

#### Disease target

Regenerative system of the brain and etiology of disease.

#### Therapeutic response to Allo

Improved functional electrical impairments *in vitro* culture of neurons from a mouse model of the disease (Cao et al., 2012).

#### Dose and treatment regimen

0.01–1 μM (*in vitro*) (Cao et al., 2012).

Preclinical studies demonstrate that Allo is efficacious in neural cells isolated from a fragile X-associated tremor/ataxia syndrome (FXTAS) mouse model (Cao et al., 2012). This mouse model has defects in neuronal morphology and migration. FXTAS related defects occur in basal electrical activity exhibited by permutation CGG repeat expansion-carrying neurons associated with a gain-of-function in type-I mGluRs and/or a loss-of-function in GABA_A_R signaling. Allo acutely improved the functional impairments as measured by electrical burst firing, in this preclinical model.

Furthermore, an Allo analog, ganaxolone, formulated in an oral suspension, given in three divided doses (ClinicalTrials.gov identifier: NCT01725152) is currently in Phase 2 proof-of-concept study in children with fragile X syndrome. Fragile X syndrome is the most common inherited form of cognitive impairment results from a single-gene disorder associated with autism. The aim of the study is to assess the safety, tolerability and efficacy of ganaxolone for treatment of anxiety and attention deficits in subjects with fragile X syndrome. The clinical trial is designed to test ganaxolone treatment compared to placebo on measures of anxiety and attention via several neuropsychological and psychometric tests.

### Diabetic neuropathy

#### Disease target

Regenerate peripheral nerve, reduce pain, increase conductivity of peripheral nerves, and etiological mechanism of disease.

#### Therapeutic response to Allo

Decreased expression of apoptosis mediators, increased nociception threshold (Afrazi et al., 2014), increased nerve conduction velocity, and restored intra-epidermal nerve fiber density (Leonelli et al., 2007).

#### Dose and treatment regimen

Intermittent exposure in rats: 5 mg or 20 mg/kg, gastric lavage, for 8 weeks (Afrazi et al., 2014); 3.3 mg/kg SC every 4 days for 8 doses; 3.3 mg/kg SC every 2 days for 16 doses (Leonelli et al., 2007).

Diabetic neuropathy is a unifying term for a heterogenous assembly of symptoms resulting from long-term glucose instability. Neuronal damage, dysfunction and apoptosis can present in patients in a myriad of ways including spontaneous pain, hypoesthesia, allodynia and hyperalegsia. Neurosteroid levels have been shown to fall in neuropathic pain conditions (Patte-Mensah et al., 2005; Saredi et al., 2005), validating the assessment of Allo for this indication. *In vivo* studies have demonstrated the effect of Allo on improving nociceptive threshold and decreased expression of apoptosis mediators (Afrazi et al., 2014). The theme of an optimal dosage regimen is continued here; Allo demonstrated superior restoration of nerve conduction velocity and intra-epidermal nerve fiber density when administered every other day for 30 days vs. every 4 days for 28 days (Leonelli et al., 2007). As in AD and MS, a higher Allo dose is not beneficial, and an *in vitro* dose-response curve study have demonstrated that the positive cell viability effects of Allo in glucose induced cell toxicity peak at the 2.5 μM dose, and decrease with increasing doses (Afrazi et al., 2014).

### Status epilepticus

#### Disease target

Spontaneous seizure activity of the brain.

#### Therapeutic response to Allo

Dampens epileptic seizure activity and reduce neuroexcitotoxicity (Rogawski et al., 2013).

#### Dose and treatment regimen

Immediate and continuous treatment regimen in 6 Hz seizure model, 1.5 mg/kg Allo IV conferred seizure protection within 1 min after dosing in mice (Rogawski et al., 2013; Zolkowska et al., 2013).

Most seizures are spontaneously terminated within a short period of time because of endogenous inhibitory mechanisms including actions of Allo on GABA_A_Rs. However, when seizures do not stop spontaneously, this results in status epilepticus, a life-threatening neurological emergency condition. Allo is currently in clinical trials on emergency basis for certain cases of status epilepticus (Rogawski et al., 2013). Previously, ganaxolone was being developed for infantile spasms (Gasior et al., 2000; Kerrigan et al., 2000; Kaminski et al., 2004). Standard treatment for status epilepticus is administration of benzodiazepines but in many cases these quickly become ineffective. GABA_A_Rs are in a continuous cycle of insertion into the cell membrane and internalization (Goodkin et al., 2005). Internalization of GABA_A_Rs occurs through clathrin-dependent endocytosis. This process is activated by calcium-phospholipid dependent protein kinase C (Chapell et al., 1998; Filippova et al., 2000) and brain-derived neurotrophic factor (Jovanovic et al., 2004). Internalization of the surface GABA_A_Rs correlates with a reduced response to GABA, whereas inhibition of internalization results in increased amplitude of synaptic GABA_A_R currents (Kittler et al., 2000).

The benzodiazepine-binding site within the GABA_A_R complex is located within the α-subunit interface with the δ-subunit and after synaptic receptor internalization, epilepsy patients often become resistant to therapy. Neurosteroid binding sites are within the α-subunit or α/β interface and are not reliant on the δ-subunit composition. Extrasynaptic GABA_A_Rs that contain a δ-subunit rather than a γ-subunit are sensitive Allo molecular targets that do not have drug resistance complications and improve treatment of seizures. Recent resurgence of interest in Allo and its analogs has prompted attention for this important disease application to dampen epileptic seizure activity and reduce neuroexcitotoxicity (Rogawski et al., 2013).

### Traumatic brain injury

#### Disease target

Glutamate excitotoxicity systems of the brain.

#### Therapeutic response to Allo

Anti-inflammatory (VanLandingham et al., 2007), neuroprotective (Sayeed et al., 2009), anti-convulsant (Rogawski et al., 2013).

#### Dose and treatment regimen

Immediate and continuous exposure. Within 8 h after injury, a continuous IV Allo regimen for traumatic brain injury (TBI) is administered during a 4-day treatment period followed by a 1-day dose de-escalation period in humans (ClinicalTrials.gov identifier: NCT01673828) (Rogawski et al., 2013).

The adult brain has been shown to possess regenerative mechanisms after infarct and injury. In rodents, proliferation in the SVZ, migration of new neurons to peri-infarct site, and survival of these new neurons has been demonstrated following stroke and involves the inflammatory cytokines and chemokines systems to aid recovery (He et al., 2004b; Djebaili et al., 2005). It has been hypothesized that angiogenesis leads to functional recovery through its interaction with one or more aspects of tissue repair, including neurogenesis (Carmichael, 2010). There is currently no effective treatment available for TBI victims. TBI may induce coma or a minimally conscious state and may lead to neurobehavioral deficits including cognitive deficits. Long-term consequences of TBI include increased risk for epileptic seizures, PD, and AD (Annegers et al., 1998; Mueller et al., 2009; Hutson et al., 2011; Johnson et al., 2012). A challenge for TBI research is the extent of heterogeneity of these brain injuries and the delayed secondary injuries due to increases in intracranial pressure, hypoxia, and glutamate excitotoxicity (Mueller et al., 2009). A TBI case study of two patients who recovered after years in a state of minimal consciousness suggests that brain regeneration and specific axonal regrowth to improve quality of life is possible even after severe TBI (Voss et al., 2006).

Allo is currently in clinical trials to assess the safety and efficacy of Allo in improving neurobehavioral outcome and reducing mortality in adults with moderate and severe TBI (ClinicalTrials.gov identifier: NCT01673828). Within 8 h after injury, a continuous IV Allo regimen for TBI is administered during a 4-day treatment period followed by a 1-day dose de-escalation period (ClinicalTrials.gov identifier: NCT01673828). This continuous treatment regimen is very different from the regenerative regimen approach taken for Allo in chronic neurodegenerative diseases. This regimen takes advantage of the anticonvulsant mechanisms of Allo action to limit acute brain excitoxicity (Figure 3). Allo and its precursor progesterone acutely reduce inflammatory cytokines after brain injury (He et al., 2004a,b; Djebaili et al., 2005). Allo has been shown to upregulate CD55, a cell surface protein that inhibits convertase enzymes to reduce neuroinflammation (VanLandingham et al., 2007). Another possible mechanism of Allo-induced neuroprotection is a direct inhibition of the mitochondrial permeability transition pore. By this mechanism, Allo was shown to inhibit calcium ion-triggered swelling in functionally intact rat liver and brain mitochondria (Sayeed et al., 2009).

## Integration of translational determinants into the clinical trial design for AD and other disorders matters

Fundamental translational determinants of success are well-defined mechanisms of action, dose-response relationships, and optimal therapeutic regimen. Mechanistically, a key to therapeutic success is an understanding of the intended activated pathways as well as those that are unintentionally activated. This is not always possible but always beneficial. A well-defined understanding of dose-response relationship includes doses that are sub-optimal and which induce toxicity or off-target effects. In the case of Allo, the dose-response relationship indicates that a sedative dose suppresses regeneration and thus establishes the maximally tolerated dose. Further, daily exposure to Allo at a non-sedative dose also suppresses regeneration. The dose and exposure relationships provide critical information for clinical trial design as well as providing key insights into the regenerative system of the brain.

The maximally tolerated dose will depend on the neurological condition, intended mechanism of action, and the therapeutic goals (Figure 3). For example, a low dose of Allo with intermittent exposure is optimal for activating regenerative responses. Whereas, TBI and status epilepticus require emergency suppression of glutamate excitotoxicity necessitating a high dose of Allo administered by continuous infusion.

Clinical trial design is inextricably linked to therapeutic success. Recently, the Food and Drug Administration (FDA) released a draft guidance document for developing drugs for early-stage AD trials (Food Drug Administration, Center for Drugs Evaluation Research, 2013) that aimed to improve the design of future AD clinical studies. The FDA guidance emphasized the use of the Clinical Dementia Rating Scale as a primary clinical scale to accelerate development of AD drugs. In addition to these cognitive and functional measures, exploratory outcomes for early stage AD trials can be incorporated into the design as secondary outcomes (Schneider, 2014). Exploratory outcomes are useful to detect responsiveness to new therapies such as correlations between neuroregenerative indicators and improvement in brain activity (Mullard, 2012, 2013; Schneider, 2014).

Adaptive clinical trials are designed to conduct interim analyses that are prospectively planned (Food Drug Administration, Center for Drugs Evaluation Research, 2010). An adaptive design affords the opportunity to modify one or more aspects of the study based on a hypothesis-driven analysis of the data. The benefit of an adaptive design is the opportunity to learn about the impact of the therapeutic agent early in the course of the study and to make course corrections earlier rather than later. These analyses would be hypothesis driven and based on predictive biomarkers of efficacy.

What defines biomarkers that align with stage of disease and are predictive of therapeutic efficacy? Potential biomarkers of efficacy relevant to regeneration could be evident as either decreased rate of degeneration or an increased rate of structural recovery. Thus we reasoned that if Allo was promoting regeneration in the brain, that regeneration could be evident in MRI-based measures of hippocampal volume, diffusion tensor imaging of white matter, and resting default mode network (Brinton, 2013). If structural integrity is related to function, then one would predict a delay in severity of dementia and or recovery of cognitive function. We anticipate, based on the temporal requirements for regeneration in the context of a degenerated brain, that the regeneration of the neural circuitry that underlies the resting default mode network has the greatest probability of being the first imaging biomarker to exhibit change over time.

Clinical trials of potential disease modifying agents require the ability to characterize a well-defined study cohort (Sperling et al., 2011a). The National Institutes of Aging and the Alzheimer’s Association published disease-staging criteria that describe the clinical stages of AD (Albert et al., 2011). For clinical development of Allo, initial targeted populations are those with mild cognitive impairment or early AD (Brinton, 2013). These populations were targeted based on preclinical analyses indicating that Allo exerted a regenerative response in transgenic AD mice with burden of pathology relevant to early stage AD. These animals exhibited cognitive and neurogenic deficits that were reversed by Allo (Brinton, 2013). At later stages of the disease, the regenerative capacity was depleted and thus not appropriate for a therapeutic that promotes endogenous regeneration (Brinton, 2013).

Collectively, it is clear that clinical trial testing of Allo across multiple neurodegenerative diseases will require translational research specific to the disease, dosing to the intended target, treatment regimen specific to the respective system, and study population specific to stage of disease.

## Concluding remarks

**Figure 5:**
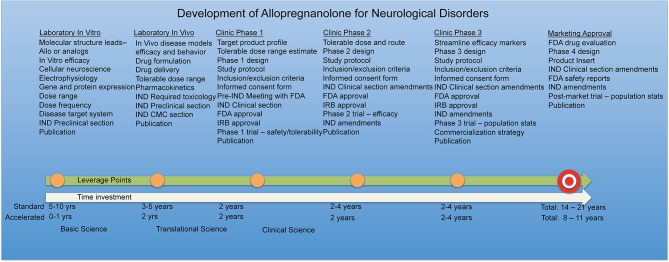
**Development of allopregnanolone for neurological disorders.** Leverage points along the standard timeline of 12 years and up to 20+ years as drug development proceeds to the marketing approval target. By sharing resources and information, it is plausible to accelerate therapeutic development timelines to 8 years or less, completing clinical trials more rapidly for unmet medical needs.

The goal of this review was to integrate existing knowledge relevant to translational development of Allo as a therapeutic agent for multiple neurological disorders. There are multiple leverage points across programs of therapeutic development that could significantly accelerate time-to-clinical trial of Allo in each of these conditions (Figure 5). Allo activates multiple systems in multiple cell types in multiple anatomical regions with therapeutic implications for multiple diseases. Each neurological disease has specific Allo dose and therapeutic regimen requirements that must be tested preclinically and must be carefully translated to clinical study design. Leverage points—such as preclinical efficacy data, preclinical toxicology data, regulatory knowledge, access to clinical-grade material, and access to clinical data matter to development of expedited timelines to utilize Allo across these disorders (Figure 5). For Allo to reach its potential as a therapeutic option, the formulation, dosing regimen, and route of administration are critical determinants of success. Considerations of gender, genetics, age, and progression of disease for both preclinical translational analyses and clinical trials are critical (Figure 4).

The therapeutic potential of Allo for multiple neurological diseases is increasingly appreciated. Different etiologies, different courses of disease progression, different mechanisms involved in the disease, will impact the therapeutic efficacy of Allo. In turn, it is essential that translational research target each of the neurological disorders and take into consideration the similarities and differences in molecular targets and etiology of disease. To suppress neuronal excitability in TBI or epilepsy, it makes sense to inhibit excitability as early as possible following injury. In other cases, maximizing GABAergic suppression of the brain in the absence of glutamate excitotoxicity is unlikely to have therapeutic benefit and could induce harm. Pragmatically, this translates into dosage levels and treatment regimens tailored to the neurological disorder and therapeutic goals in the context of the relevant systems biology.

We have reviewed herein compelling preclinical discovery outcomes that strongly suggest the therapeutic potential of Allo. Translation of Allo to clinical studies requires systematic establishment of the optimal target engagement, dose, treatment regimen, duration of treatment, and safety. What remains is to leverage the discovery science into the path of translation. Fortunately, and remarkably, there are multiple opportunities to leverage existing translational knowledge and to systematically apply that knowledge in disease relevant models to accelerate determination of Allo efficacy across multiple disease states.

## Conflict of interest statement

Patents pending on allopregnanolone as a therapeutic for mild cognitive impairment and Alzheimer’s disease.

## Footnotes

^1^http://www.fda.gov/drugs/drugsafety/ucm334033.htm
